# Carbon Isotope Fractionation Characteristics of Normally Pressured Shale Gas from the Southeastern Margin of the Sichuan Basin; Insights into Shale Gas Storage Mechanisms

**DOI:** 10.3390/nano13010143

**Published:** 2022-12-28

**Authors:** Changyu Yang, Chenjun Wu, Qilin Xiao, Xu Zhang, Juan Teng, Jiaxin Li

**Affiliations:** 1 College of Resources and Environment, Yangtze University, Wuhan 430100, China; 2 SINOPEC East China Branch Company, Nanjing 210019, China

**Keywords:** normally pressured shale gas, Longmaxi Formation, pressure system, carbon isotope, preservation condition

## Abstract

Since the development of shale gas in the Wufeng–Longmaxi Formation in the Sichuan Basin, China’s shale gas production and reserves have increased rapidly. The southeastern margin of the Sichuan Basin is located in a normally pressured transition zone, where single well gas production varies greatly under complex geological structures. In order to reveal the shale gas enrichment mechanism and favorable shale gas regions, shale gas samples from production wells were collected from different structures, with the formation pressure coefficient ranging between 0.98 and 1.35. The gas components and carbon isotope characteristics of normally pressured shale gas were investigated. The carbon isotope characteristics of the Wufeng–Longmaxi shale gas from the basin scale was mainly controlled using thermal maturity; as the thermal maturity increased, heavier carbon isotopes were found, in addition to drier shale gas. For normally pressured shale gas, the composition of δ^13^C_1_ and δ^13^C_2_ becomes heavier, and the dryness coefficient decreases with the decreasing pressure coefficient; this is not consistent with the results from thermal evolution. By comparing possible influencing factors, it is evident that the change in geological structure destroys the original shale gas reservoir, which leads to the escape of some gases, and it may be the main factor that contributes to the gas geochemical characteristics of the normally pressured shale gas. Compared with the geological parameters of the shale samples, such as mineral composition, organic abundance, organic pore distribution, and gas content, the carbon isotope characteristics of normally pressured shale gas show a higher efficiency, thus indicating favorable sweet spot evaluations for shale gas in the studied areas.

## 1. Introduction

Shale gas is an unconventional hydrocarbon, and shale gas reservoirs have attracted increasing attention in recent years with respect to global oil and gas exploration. These studies have been driven by United States-led breakthroughs that have improved its potential for exploitation [1,2,3]. China contains abundant shale gas resources, which are estimated to exceed 100 × 10^12^ m^3^ in geological reservoirs [4]. The development of shale gas within the Wufeng–Longmaxi Formation in the Sichuan Basin has helped to rapidly increase China’s shale gas production and total number of reserves [1].

The Wufeng–Longmaxi Formation in the Sichuan Province is a potential shale gas reservoir. The lithologies have experienced multiple tectonic events since their deposition, which have generated complex structural relationships between individual units. This set of strata is one of the most important sources of rock horizons for marine shale gas exploration in southern China. The Sichuan Basin has a stable internal structure, and its faults are underdeveloped; this enhances the likelihood of shale gas being preserved. Indeed, preliminary exploration has shown that the basin likely contains rich reserves. Since 2010, commercial shale gas has developed in the Weiyuan, Jiaoshiba, Fushun-Yongchuan, Changning–Zhaotong, and Weirong areas within the basin, along with other regions. Furthermore, several national shale gas demonstration areas have been established in the basin, which has heralded a new era of large-scale shale gas development across China. The enrichment and production of large volumes of shale gas requires the development of particular geological conditions in the subsurface environment; thus, studying the variables that affect shale gas preservation is of great significance for the sustainable development of shale gas resources in China [5,6,7,8,9].

Shale gas reservoirs that are situated along the margin of the basin have undergone different degrees of structural transformation from those outside the basin. Although commercial shale gas has been developed in Nanchuan, Wulong, and other areas, complex geological structures, low rates of shale gas production, and the high cost of development have all hindered large-scale commercial development [10,11]. Much research has been conducted with regard to determining the conditions under which shale gas is preserved underground, and these studies have shown that the morphological characteristics of shale pores likely reflect differences in preservation conditions due to differences in structural strength. Shale organic matter pores that have high preservation potential are spherical and similarly sized, whereas organic matter pores that have poor preservation potential are slit-shaped and have highly variable sizes [12,13,14]. The permeability of shale is mainly affected by its porosity [15]. Furthermore, both the geological characteristics of the roof and floor lithologies, and the type of contact between the roof and floor lithologies in a shale gas reservoir, strongly influence the preservation conditions of gas-bearing shale. Low-porosity roof and floor units can reduce the outward migration of shale gas, which allows it to be effectively preserved [16,17]. Post-formation tectonic deformation can also affect the preservation potential of shale gas; for example, by affecting reservoirs via uplift and denudation, fault motion, and structural deformation. In addition, shale gas preservation is promoted with high pressure coefficients. When the self-sealing property of shale is preserved, organic-rich shales theoretically develop extremely high excess pressures, and the pressure coefficient in a sample is often positively correlated with its gas content [16,18]. These studies represent landmark contributions towards understanding shale gas preservation conditions, and they have provided valuable geological data related to oil and gas formation that have improved China’s actual shale gas exploration; however, they cannot assist with guiding exploration for shale gas in structurally complex areas. Studying the relationship between carbon isotope values and tectonic structures can provide an improved theoretical understanding for practical shale gas exploration.

Carbon isotope values play an important role in shale gas exploration [19]. Shale gas reservoirs differ from conventional gas reservoirs by virtue of shale gas being trapped within the same reservoir in which it was generated; as such, the carbon isotope values of shale gas tend to show clear vertical trends within the formation. Alkane carbon isotope reversal is common in high-maturity shale gas fields, and the amplitude of such a reversal is often positively correlated with shale gas content. This characteristic can thus be used to locate the highest concentration of shale gas within a reservoir [20,21,22,23]. At present, our understanding of how carbon isotope values in a shale gas reservoir correlate with structural characteristics is incomplete. In order to study how carbon isotopes fractionate under different structural conditions, we sampled and analyzed shale gas from development wells in different structural areas within the Sichuan Basin, at the edge of the basin, and outside the basin. Such work is critical for determining the relationships between the carbon isotope fractionation of shale gas and structures within a reservoir, which, in turn, can provide new guidelines for the more extensive and efficient exploration of regions rich in shale gas.

## 2. Geological Background

The Sichuan Basin, Southwest China, is a large intercontinental sedimentary basin that developed on Paleozoic marine strata, and it exposes large areas of shale [1,24]. The Sichuan Basin has a complex tectonic and sedimentary history, as it is located on the upper part of the Yangtze Platform, which has experienced Caledonian (Late Sinian–Silurian), Hercynian (Devonian–Permian), Indosinian (Triassic), Yanshanian (Jurassic–Cretaceous), and Himalayan (Tertiary–Quaternary) deformation. During the Jinning tectonic event, the pre-Sinian geosyncline was uplifted and folded, and the Yangtze craton basement became consolidated. Basement rocks in the basin were formed later during the Chengjiang tectonic event, with the subsequent generation of the Chengdu and Kangdian crust occurring between the Late Ordovician and the Early Silurian periods. This episode of crustal growth significantly changed the tectonic architecture of the Sichuan Basin, causing it to transition from a passive continental margin into a foreland basin; this is evident upon examination of the sedimentary rocks which form a singular topographic depression (i.e., basin) surrounded by three uplifted regions [24,25]. Before the Yanshanian deformation event, tectonic activity in the Sichuan Basin was dominated by subsidence and uplift; however, after this event, tectonic activity was dominated by intensive lateral compression, which formed several fold belts [9,20,26,27].

**Figure 1:**
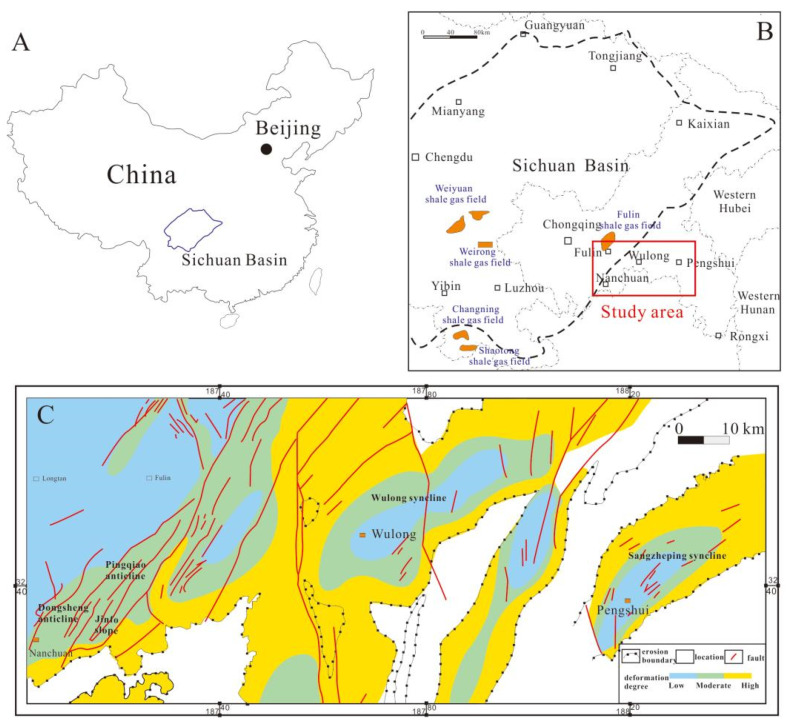
Location and geologic deformation degree distribution of the studied area in the southeastern margin of the Sichuan Basin, China. (**A**). Map of China. (**B**). Geological map of Sichuan Basin. (**C**). Geological map of the studied area.

The study area is located in the southeastern Sichuan Basin between 106°54′ E and 107°27′ E, and 28°46′ N and 29°30′ N, which is southeast of the Chongqing Basin edge transition zone, and it lies within the transition zone between the Sichuan Basin and an external composite fold belt. The study area is adjacent to the western Hunan–Hubei Fault zone, the Qiyueshan Fault zone, the northern Jiaoshiba structure, the Nanchuan–Zunyi Fault zone, and the Jinfoshan uplift in the south, and it includes Nanchuan, Wulong, Pengshui, and other counties and cities. The terrain is dominated by mountains that have escarpments sloping from southeast to northwest. This topography is controlled by the Qiyueshan Fault, which is a compressional–torsional strike-slip fault that trends from north to south. The structural deformation associated with this fault is more intense than elsewhere within the Sichuan Basin, and tectonic activity away from the fault generally occurs towards the northeast. The area exposes an Early Silurian continental shelf and associated low-lying areas, which has accumulated thick sequences of organic-rich shale [28,29]. The study area also lies within a transition zone between rocks that formed under high and normal fluid pressures, although most shales contain normally pressured shale gas (Figure 1). The area has experienced many tectonic deformation events, although the Caledonian, the Hercynian, the Indosinian, and the Yanshan–Himalaya events had the greatest influence on the region’s structural evolution. In particular, the Yanshanian and Himalayan deformation events were the most intense, and they impacted the broadest areas of the southeastern Sichuan Basin [1,30].

**Figure 2:**
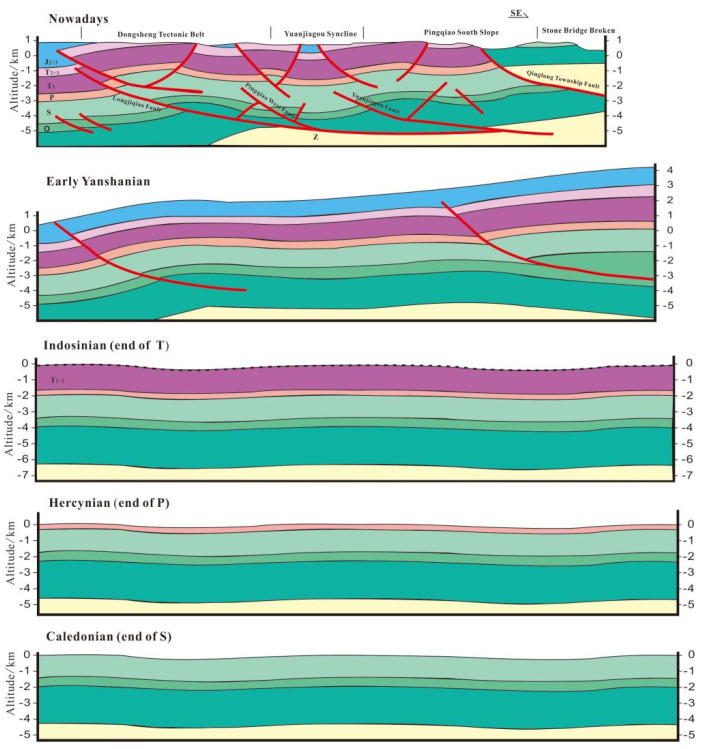
Tectonic movement of the studied areas during the Late Yanshan–Himalayan period.

The Caledonian, Hercynian, and Indosinian events were associated with relatively weak tectonic activity on the Yangtze platform, as it mostly experienced vertical uplift and subsidence at those times. Large-scale uplift and erosion led to a period of isostatic quiescence that is recorded in the geology of the region by largely undeformed strata. During the Yanshan–Himalayan period, the study area was subjected to northwest-directed compression against orogenic belts in the southeast, driven by the subduction of the Indian plate beneath the Asian plate. Broad-scale strata uplift caused widespread weathering and erosion, which formed a “barrier-type” structure with alternating residual anticlines and synclines across the region with NE–SW orientations. Shales in the Wufeng–Longmaxi Formation began to generate hydrocarbons during the Late Permian (P_2_) period, and they reached their maximum depths during the Early Jurassic–Late Cretaceous (J_1_–K_2_) periods. The continued uplift of strata during the Late Yanshan–Himalayan period led to the complete denudation of the Wufeng–Longmaxi Formation shale in some areas (Figure 2). Subsequent structural deformations in the study area were more intense than in the area around Fuling, which led to significant differences in preservation conditions, gas occurrence, and coefficients of formation pressure in each region. The tectonic uplift in the Pengshui–Nanchuan area decreases in age from east to west, although the intensity of deformation weakens in the same direction. Furthermore, the duration of later deformations, and the dissipation of shale layers, lessens from east to west. Moreover, the formation’s stratigraphy becomes more complete, and the thickness of high-quality shale increases. Indeed, shale gas has the highest preservation potential near the Sichuan Basin, which has allowed widespread shale gas enrichment in the area [11,26,31,32,33].

## 3. Samples and Methods of Analysis

To study the carbon isotope fractionation characteristics of normally pressured shale gas in the southeastern margin of the Sichuan Basin, we collected 16 shale gas samples from industrial production gas wells located in the Nanchuan, Wulong, and Pengshui areas. The shale gas samples were collected from different structural positions in systems that formed under varying pressures. Five samples with a formation pressure coefficient larger than 1.35 were collected from the Pingqiao anticline. Three samples with formation pressure coefficients in the range of 1.20–1.35 were collected from the Dongsheng anticline. Three samples with formation pressure coefficients of 1.12–1.20 were collected from the Jinfo slope structure. Finally, two shale gas samples with formation pressure coefficients of 0.98–1.08 were collected from the Wulong and Sangzheping synclines.

The molecular composition of generated gas was analyzed using a MAT271 mass spectrometer–continuous flow gas chromatograph (GC) system (Finnigan MAT Company, Waltham, MA, USA). The carbon isotope characteristics of the generated hydrocarbon gases were measured using a GC (Agilent 6890, Agilent Technologies, Palo Alto, CA, USA) coupled with an isotope ratio mass spectrometer (IRMS; Finnigan Delta plus XP, Thermo-Fisher, Bremen, Germany) via a combustion interface (GC Combustion III). The operating temperature program was 30 °C for 3 min, followed by a 10 °C/min increase to 250 °C; the samples were held at this temperature for 50 min. Three pulses of standard pure CO_2_ gas, pre-calibrated against an inter-laboratory recognized reference of CO_2_, were injected via the GC-C III interface to the IRMS for the calculation of δ^13^C values. The average reproducibility of the GC–IRMS analysis was better than ±0.3‰ for natural gas standards [34].

## 4. Results and Discussion

### 4.1. Influence of Thermal Evolution Characteristics on Shale Gas Geochemistry

**Figure 3:**
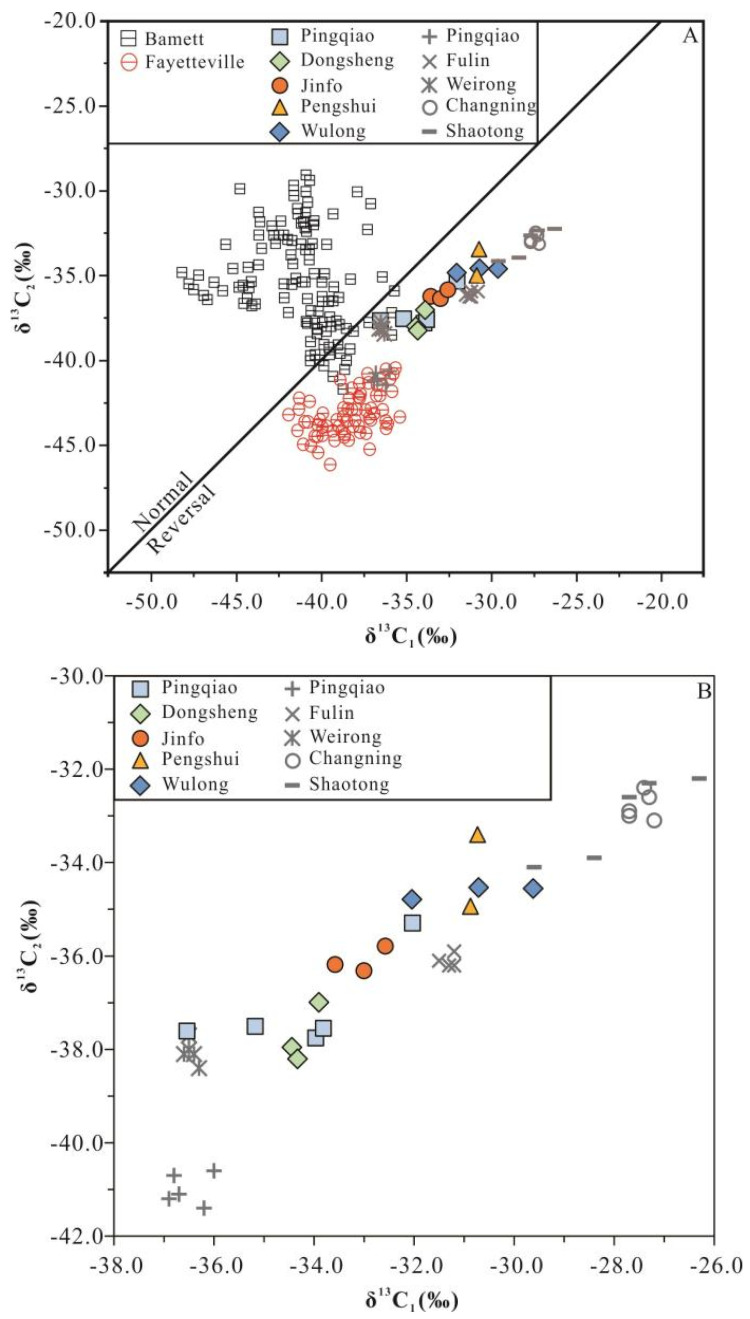
Plots of δ^13^C_1_ vs. δ^13^C_2_ for shale gas samples worldwide (**A**) and the studied area (**B**). Data concerning the Barnett and Fayetteville shale gases are from Zumberge et al. [23]; data concerning the Longmaxi shale gas in the Weiyuan and Weirong Field are from Dai et al. [38,39,40]; data concerning the Fuling Field are from Li et al. [40,41,42,43,44]; and data concerning the Changning field are from Dai et al. [38,40,45].

The shale gas components of the Wufeng–Longmaxi Formation, sampled from the Weiyuan, Jiaoshiba, Changning, Weirong, Nanchuan, Wulong, and Pengshui shale gas-producing areas in the Sichuan Basin, are dominated by methane (95.52–99.45%, with an average of 98.16%). Heavy hydrocarbons, such as ethane, occur in low concentrations, and their abundance decreases proportionally to the increase in their number of carbon atoms. For example, ethane comprised approximately 0.09–0.99% of the total gases measured, with an average of 0.52%; propane comprised approximately 0–0.1%, with an average of 0.016%; and butane and heavier hydrocarbons were absent altogether. Measured carbon isotope values of methane were between −37.3‰ and −26.7‰, with an average of −31.3‰, and measured ethane carbon isotope values were between −42.8‰ and −31.6‰, with an average of −35.3‰. The dryness coefficient (C_1_/C_1–5_) was very high, with a total range of 99.19–99.55%, and average values in each region exceeded 99%. The non-hydrocarbon content of the analyzed samples was low, making them typical dry gases, and the carbon isotope values of alkane gas (C_1–3_) were partially or completely reversed (Figure 3A).

Wang et al. [35] believed that the Longmaxi Formation in the Sichuan Basin mainly underwent secondary cracking and Rayleigh fractionation; however, the dominant factor changed from secondary cracking to Rayleigh fractionation with increased maturity. In that work, four causes of carbon isotope reversal in marine facies shale in the Sichuan Basin were documented: (1) the combination of kerogen thermal degradation and crude oil pyrolysis to natural gas; (2) Rayleigh fractionation effects; (3) and mixing of oil-formed gas and coal-formed gas. Cao et al. [36] pointed out that secondary cracking of liquid hydrocarbons may have caused an inverse correlation between carbon isotope values and carbon numbers. Concurrently, late-stage structural deformation, uplift, and differences in geochemical conditions may have generated different carbon isotope compositions between regions in the study area. Wang et al. [37] reported that the carbon isotope reversal that was documented in Longmaxi Formation shale gas in the Sichuan Basin was mainly due to the mixing of homologous gases produced during different periods, such as crude oil cracking gas and kerogen cracking gas. Previous studies have suggested that increased thermal maturity can partially reverse the carbon isotope composition sequence of shale gas, or even completely reverse it during late-stage heating. Indeed, some studies have reported general reversals of carbon isotope values from the Wufeng–Longmaxi Formation in the Sichuan Basin, with values of δ^13^C_1_ > δ^13^C_2_ > δ^13^C_3_ or δ^13^C_1_ > δ^13^C_2_ < δ^13^C_3_; although, the magnitude of reversals varies between regions [20,23]. Xia et al. [22] proposed a four-stage model describing how δ^13^C varies according to sample maturity. The initial low maturity stage involves almost no liquid hydrocarbon; however, as maturity gradually increases during the second stage, the volume of gas generated from liquid hydrocarbons also increases, although ethane carbon isotope values decrease. During the third stage, when maturity further increases, a reversal between δ^13^C_1_ > δ^13^C_2_ occurs. Maturity continues to increase during the fourth stage, making the samples highly over-mature. At this stage, the liquid hydrocarbon has been almost exhausted, the contribution of liquid hydrocarbon secondary gas has been reduced, and carbon isotope values should return to a normal sequence.

As shown in Figure 3B, we noted significant differences in gas geochemical characteristics of different shale gas-producing areas in the Sichuan Basin. The average methane contents in the Weiyuan and Weirong areas were 97.86% and 96.58%, respectively, which were lower than those in the Changning (98.56%) and Jiaoshiba (98.32%) areas. The Changning area recorded the highest methane content, although it had lower ethane and propane contents than the Weiyuan and Jiaoshiba areas. In terms of isotope characteristics, the δ^13^C_1_ and δ^13^C_2_ values in the Weiyuan area were significantly lower than those in the Changning and Jiaoshiba areas, and the Changning area recorded the heaviest methane and ethane contents compared with all other analyses.

Previous studies have suggested that the carbon isotope characteristics of shale gas are affected by the degree of thermal evolution, with alkane gas characteristics becoming gradually heavier during heating [46,47,48,49,50,51]. The Wufeng–Longmaxi Formation in the Sichuan Basin records a high overall thermal maturity; for example, the vitrinite reflectance (*Ro*) distribution in the lower part of the Lower Silurian was approximately 1.25–3.33%, with an average of 2.45%, thus indicating that it reached the late mature to over-mature stage. The *Ro* of the Upper Ordovician was 2.22–3.59%, with an average of 2.89%, which represents the over-mature stage [52,53]. The following *Ro* values were reported for shale samples: approximately 2.0–2.2%, in the Weiyuan area; an average of 2.1% in the Weirong area; approximately 2.5–2.6% in the Pengshui area; approximately 2.4–2.8% in the Jiaoshiba area; approximately 2.6–2.7% in the Nanchuan area; approximately 2.6–2.7% in the Wulong area; and approximately 2.8–3.3% in the Changning area. These values indicate that the shales are highly mature and over-mature in terms of their evolutionary stages, and they are mainly generating dry gases [45,54,55,56,57]. Shales in the Weiyuan area have a slightly lower maturity than other areas, their humidity is relatively high, and the carbon isotope composition of shale gas is generally light. The Changning and Zhaotong areas show the highest degree of thermal evolution compared with other areas in the Sichuan Basin, and the shale gas has the highest methane content and heaviest carbon isotope composition. The *Ro* value of shale in the study area lies between values for the Weiyuan and Changning areas.

Highly mature shale is amenable for the secondary cracking of oil and gas, which leads to the secondary cracking of shale gas; this occurs at relatively great depths below the Earth’s surface. During oil and gas cracking, alkanes with large molecular weights have relatively small activation energies required for the cracking process to proceed, which makes the cracking process easier as the chemical bonds of light isotopes are easier to break. Residual heavy isotope macromolecules are cracked during the later stages of oil and gas cracking, allowing alkanes with low molecular weights to gradually become enriched in heavier isotopes during their evolution [36,46,51]. Values for δ^13^C_1_ and δ^13^C_2_ therefore increase, reflecting a greater maturity. Studies have shown that the dryness coefficient (D = C_1_/C_1–5_) or humidity coefficient (W = C_2–5_/C_1–5_) of natural gas is closely related to its hydrocarbon source rock. During the pyrolysis of organic matter, the dryness coefficient of natural gas increases as organic matter becomes more thermally evolved. Many researchers therefore use the dryness coefficient or humidity coefficient of natural gas to measure the maturity of natural gas [22,58,59]. Dai et al. [60] summarized the relationship between organic vitrinite reflectance and the humidity coefficient as *Ro* = −0.419lnW + 1.908. Shale in the Changning and Jiaoshiba areas showed a higher degree of thermal evolution than in the Weiyuan area, and the carbon isotope values of methane and ethane in the Weiyuan area were significantly lower. With regard to the whole Sichuan Basin, carbon isotopes in the Weiyuan, Jiaoshiba, and Changning areas became heavier with increased thermal maturity, alongside an increased dryness coefficient.

### 4.2. Factors That Control Gas Geochemical Characteristics

**Figure 4:**
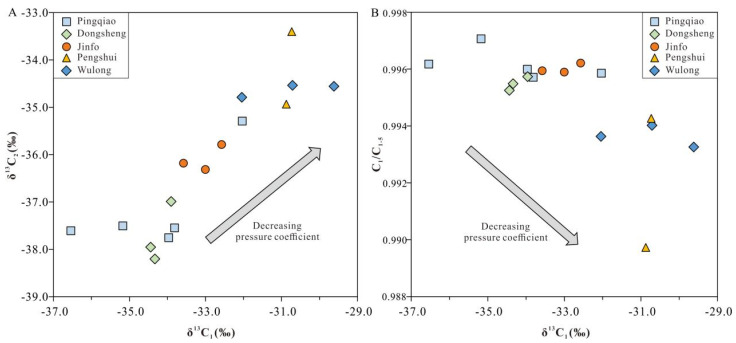
Plots of (**A**) δ^13^C_1_ vs. δ^13^C_2_ and (**B**) δ^13^C_1_ vs. C_1_/C_1-5_.

The overall pressure coefficients reported in the Weiyuan, Jiaoshiba, and Changning shale gas-producing areas were relatively high, which shows that the shale gas reservoir is overpressured and that gas preservation conditions in these areas are ideal for production; however, the shale gas-producing regions of the Nanchuan, Wulong, and Pengshui areas considered in this study contain atmospheric shale gas with a relatively low pressure coefficient, mainly within the range of 0.98–1.35. The degree of thermal evolution of the Longmaxi Formation in the Nanchuan, Wulong, and Pengshui areas is similar, with values in the range of 2.5–2.7%; although, their gas carbon isotope values and dryness coefficients are quite different (Figure 4). The C_1_/C_1–5_ values of units from the Pingqiao anticline, Dongsheng anticline, Jinfo slope, Wulong syncline, and Sangzheping syncline in the study area gradually decrease. If the conditions outlined above are not satisfied, the *Ro* value also gradually decreases, and the δ^13^C_1_ and δ^13^C_2_ compositions gradually become lighter; however, our data show that the δ^13^C_1_ and δ^13^C_2_ compositions in these five structural areas gradually became heavier as the dryness coefficient decreased, which does not conform to the abovementioned relationship. Although the degree of thermal evolution is an important factor that affects carbon isotope fractionation, the above anomalies indicate that the degree of thermal evolution may not be the most important control with regard to the differences between δ^13^C_1_ and δ^13^C_2_ for normally pressured shale gas in the study area. Consequently, the variations in carbon isotopes and gaseous components of shale gas in the southern margin of the Sichuan Basin may have been caused by alternative factors.

The observed reversal of methane and ethane carbon isotope signatures in shale gas may have been caused by a thermochemical sulfate reaction (TSR), the microbial degradation of alkane gas, different types of hydrocarbon-generating parent materials, the mixing of organic and inorganic alkane gas, and/or the mixing of oil-formed gas and coal-formed gas.

Different types of parent materials have different effects on carbon isotopes during hydrocarbon generation and expulsion; indeed, the carbon isotope characteristics of alkanes are also affected by the characteristics of their parent materials [40,61,62,63]. The Wufeng–Longmaxi Formation in the Sichuan Basin hosts a complete set of marine sediments; therefore, the hydrocarbon parent materials for all areas were relatively similar, and were mainly algae and bacteria. As such, the effects that different hydrocarbon parent materials have on carbon isotope characteristics can be excluded from consideration [58,64].

Previous studies have shown that the δ^13^C_2_ of oil-formed gas is lighter than that of coal-formed gas under identical or similar evolutionary conditions. Ethane carbon isotopes are most commonly used to effectively index and distinguish coal-formed gas from oil-formed gas; more specifically, previous work has shown that oil-formed gas has δ^13^C_2_ < −29.0‰ [65,66,67,68]. The measured δ^13^C_2_ in the study area was less than −29‰, which indicates that the studied samples are oil-formed gas. Inorganic genetic gas has carbon isotope series characteristics of δ^13^C_1_ > δ^13^C_2_ > δ^13^C_3_, and the lower limit of δ^13^C_1_ for inorganic genetic gas ranges between −20‰ to −30‰ [69,70]. The alkane gases in the study area do not have this feature, thus indicating that none are inorganic in origin, which is in accordance with Huang [67], who proposed that each layer of natural gas in the Sichuan Basin is organic. In addition, shale gas in the Wufen–Longmaxi Formation is self-generated and self-stored, thus indicating that there has been no mixing between organic and inorganic alkane gases, or between coal-formed and oil-formed gases.

The process of TSR refers to sulfate minerals being reduced to sulfides using hydrocarbons and a variety of thermodynamic driving forces, with hydrocarbons being simultaneously oxidized. This reaction accelerates the cracking of hydrocarbons or crude oil. In this reaction, the ^12^C–^12^C bond is the first to break, such that ^12^C is more involved in the reaction and ^13^C is preferentially retained in hydrocarbons; therefore, ^13^C becomes relatively enriched in residual hydrocarbons after the reaction is finished, which causes the isotope to become heavier. The carbon isotope weight gain in heavy hydrocarbons is greater than that of CH_4_; thus, as heavy hydrocarbons preferentially participate in the reaction, the dryness coefficient increases commensurately. The TSR reaction cannot reverse the carbon isotope characteristics of methane and ethane; however, it can return a reversed carbon isotope profile to normal [58,71,72]. Therefore, we conclude that the carbon isotope anomalies of methane and ethane in the study area were not caused by TSR.

Studies have shown that microbial degradation decreases propane content and causes a heavier carbon isotope composition, accompanied by an increase in methane content and a lighter carbon isotope. Microorganisms preferentially degrade propane, which causes the carbon isotope of the remaining hydrocarbons to become heavier. Gases of bacterial origin are typically rich in methane and they have lighter isotope signatures than thermogenic gases [71,73,74]; therefore, the effect of microbial degradation on the methane isotope value in the study area can be excluded.

Structural modification may lead to a reversal of carbon isotope trends. During tectonic uplift, ^12^C-rich methane will preferentially diffuse out of a system, followed by ^12^C-rich ethane and propane, which causes residual methane to be more enriched in ^13^C than residual ethane and propane. It has been postulated that geological deformation events can destroy an original shale gas reservoir, thus allowing some gases to escape and generate a reversed carbon isotope signature in the remaining gas.

The tectonic evolution of the Wufeng–Longmaxi Formation has significantly differed in various parts of the southeastern Sichuan Basin, which has led to an uneven preservation of its strata. Here, we compared the Pingqiao anticline in the Nanchuan area, the Pingqiao anticline, the Jinfo slope, the Wulong syncline in the Wulong area, and the Sangzheping syncline in the Pengshui area as examples of different deformational histories. The southeastern margin of the Sichuan Basin experienced an extended period of tectonic deformation, including a NW-trending compression during the Yanshan–Himalayan period that is associated with a subduction of the Indian plate beneath the Asian plate. Uplifted strata were denuded, forming an extensive fold structure with alternating residual anticlines and residual synclines that show evidence of progressive deformation from east to west. Structural uplift in the Pengshui–Nanchuan area occurred later, in a progressive manner, from east to west, and deformation-related structures became less pronounced. Structural deformation features vary from closed synclines to wide synclines, as well as basin margin slopes and anticlines [31,33].

Structural uplift in the Sichuan Basin began at ~89 Ma, and it has continued to the present day in the Nanchuan area. The amount of uplift-related denudation in topographically positive areas, such as the Pingqiao anticline and the Dongsheng anticline, was ~2500–3700 m. The formation pressure coefficient within the Dongsheng anticline was 1.20–1.35, and the formation pressure coefficient within the Pingqiao anticline was greater than 1.35. The Jinfo slope is relatively well formed, the regional caprock, roof, and floor have been strongly sealed, the structure is simple and stable, and it shows a relatively mild degree of deformation. In addition, the Tongtian Fault does not affect the structure, and the edge-control fault is also sealed. The upward direction of the slope structure has developed reverse-fault shielding, which blocked a potential shale gas escape channel, which limited the total amount of shale gas that could migrate to the denuded area. Instead, shale gas was largely retained in the footwall [75], which promoted its preservation.

A formation pressure coefficient between 1.0–1.2 allows for high rates of gas production. The Pengshui and Wulong areas outside of the Sichuan Basin experienced early structural uplift, extensive deformation, and the considerable denudation of strata. The Sangzheping syncline experienced structural uplift at about 135 Ma, and it has continued to the present day, with uplift-related denudation determined to have occurred at approximately 3500–5000 m. Uplift in adjacent anticlinal structures caused denudation of more than 6500 mm with associated formation pressure coefficients of 0.98–1.08. The structural uplift of the Wulong syncline began at about 90 Ma, with associated uplift-related denudation at approximately 1500–4000 m. Uplift-related denudation in adjacent anticlines was approximately 5000–6000 m with associated formation pressure coefficients of 1.05–1.1. The Wufeng–Longmaxi Formation has remained in the syncline, and shale gas has been diffused and has dissipated for an extended period of time; however, preservation conditions are poor and gas production has been low. The Jiaoshiba structure within the Sichuan Basin therefore represents a typical overpressured shale gas area. Structural uplift in this locality began at ~85 Ma, it has continued to the present day, and there has been ~3500–4000 m of uplift-related denudation; although, the Wufeng–Longmaxi Formation is not exposed at the surface. As such, shale gas in this region can escape relatively quickly over small length scales, which are favorable conditions for preservation. In this region, the formation pressure coefficient was ~1.55 and shale gas production has been high [56].

This comparison of tectonic structures shows that the pressure coefficient gradually increases as one moves from outside to inside of the Sichuan Basin, as the age of tectonic uplift shifts from earlier to later periods, as the preservation conditions of shale gas improve, and as shale gas production gradually increases. By contrast, preservation conditions recorded in the Nanchuan–Pengshui area of the study region gradually deteriorated over time, as shown by carbon isotopes becoming heavier and the dryness coefficient gradually decreasing; this indicates that the structure lost some gases. By comparing and analyzing differences between the tectonic evolution of the samples, we can infer that the variable distribution of carbon isotopes and gaseous components of atmospheric shale gas in the southern margin of the Sichuan Basin was mainly affected by the partial escape of gas, which is caused by region-specific structural variations.

### 4.3. Insights into the Enrichment Mechanisms of Normally Pressured Shale Gas and Regions of Favorable Formation

The mechanisms that enrich normally pressured shale gas, and techniques used for exploration in tectonically complex regions, have been widely discussed in recent studies [76,77]; although, most work has considered mineral compositions, organic abundances, organic-pore distributions, and gas contents. The mineral compositions and organic abundances of favorable shale gas layers in the Wufeng–Longmaxi Formation were similar due to widely distributed organic-rich shales across the studied regions; these were deposited in a common sedimentary environment [10,78,79]. He et al. [10] suggested that high-quality carbon-, silicate-, and graptolite-rich shales formed on a deep-water continental shelf are the source rocks for shale gas enrichment.

**Figure 5:**
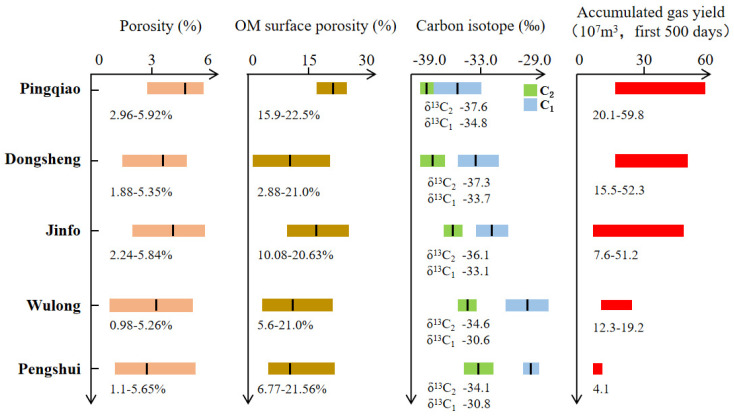
Distribution of porosity, OM (organic matter) surface porosity, carbon isotope (δ^13^C), and accumulated gas yield for shale gas systems in the studied areas.

Organic pores are a primary reservoir space for shale gas storage in the Wufeng–Longmaxi Formation in the Sichuan Basin. Studies of the distribution of organic pores in the studied area have shown that shales of the Wufeng–Longmaxi Formation vary in terms of pore space, the sizes of which correspond with their pressure coefficient during formation [80,81]. In general, organic pores in more highly pressured regions are more favorable for gas storage, as they have a better surface porosity, roundness, and pore diameter. In addition, favorable shale gas layers in highly pressured regions show more homogeneous porosity and organic pore parameters, thus indicating a more consistent pore distribution, as shown in Figure 5. As the pressure coefficient decreases from the northwest to southeast of the basin margin’s transition zone, organic pores in shale are more complex, with a lower roundness and surface porosity. For the Wulong and Sangzheping synclines, organic-rich shales located at different depths on the syncline limbs show very different pore characteristics. More specifically, shales have a higher porosity and better organic pore distribution at greater depths, which indicates a higher potential for shale gas preservation.

**Figure 6:**
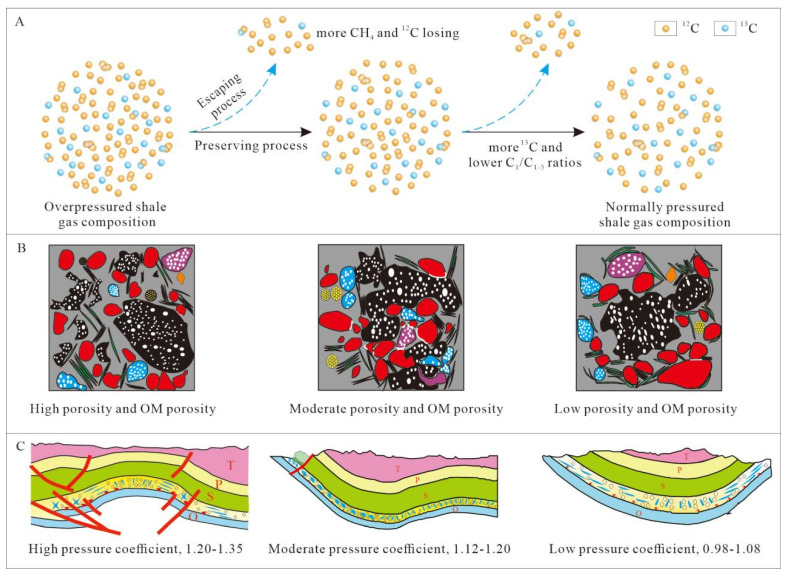
Schematic diagram of the variation in carbon isotope compositions (**A**) and pore distributions (**B**) for shale gas systems during geologically long-term preservation and loss processes under different structural conditions with various pressure coefficients (**C**). Letters T, P, S, O represent Triassic, Permian, Silurian, and Ordovician stratas.

Alongside the abovementioned geological characteristics of the shale samples, shale gas geochemistry (i.e., shale gas components and carbon isotope signatures) can be used to effectively discriminate between shale gas samples collected from various structural positions. Figure 5 shows the carbon isotopes of methane and ethane from shale gas samples collected from different structural positions, alongside their variable pressure coefficients. As discussed above, changes in the carbon isotope trend suggests that shale gas preservation conditions improved along the Sangzheping and Wulong synclines (i.e., with low pressure coefficients) towards the Dongsheng and Pingqiao anticlines (i.e., with high pressure coefficients). Changes in carbon isotopes are consistent with changes to shale gas preservation conditions and shale gas yields, as shown in Figure 5. Based on the abovementioned study, the schematic diagram of the variations between carbon isotope compositions and pore distributions, for shale gas systems during geologically long-term preservation and loss processes, under different structural conditions, with various pressure coefficients, is presented in Figure 6. As the pressure coefficient decreases as we move from anticline structures to syncline structures, the shale gas system lost more CH_4_ and ^12^C during the process wherein gas escapes, with the pore system becoming worse. Notably, the Dongsheng and Pingqiao anticlines, which are associated with high pressure coefficients, recorded a much higher shale gas yield in the first 500 days of production. Carbon isotope characteristics of normally pressured shale gas from the southeastern margin of the Sichuan Basin can therefore be used as indicator for favorable shale gas ‘sweet spots’ when performing evaluations of these areas.

## 5. Conclusions

We investigated the carbon isotope characteristics of normally pressured shale gas from the Dongsheng and Pingqiao anticlines, the Jinfo slope, and the Sangzheping and Wulong synclines situated at the southeastern margin of the Sichuan Basin. Furthermore, we discussed the factors that control gas geochemical characteristics, and we have provided insights into normally pressured shale gas enrichment mechanisms and favorable regions for its accumulation. Several conclusions can be drawn from this research.

(1) The carbon isotope characteristics of shale gas in the Wufeng–Longmaxi Formation in the Sichuan Basin are mainly controlled by thermal maturity. The carbon isotope values of shale gas become heavier with increasing thermal maturity; for example, lighter δ^13^C_1_ and δ^13^C_2_ values were documented in Weiyuan, but heavier δ^13^C_1_ and δ^13^C_2_ values were documented in Changning.
(2) The shale gas samples collected from various localities in the study region record a similar thermal evolution; however, they show different geochemical characteristics. The composition of δ^13^C_1_ and δ^13^C_2_ became heavier across five studied structural areas, in accordance with a decrease in the pressure coefficient, whereas the dryness coefficient of the studied shale gas decreased, as did the decreasing pressure coefficient. This indicates that the degree of thermal evolution was unlikely to be the main factor causing the differences between δ^13^C_1_ and δ^13^C_2_ in normally pressured shale gas in the study area.
(3) Alternative factors that control the differences between δ^13^C_1_ and δ^13^C_2_ in normally pressured shale gas were discussed. We determined that factors such as TSR, the microbial degradation of alkane gas, different types of hydrocarbon-generating parent materials, the mixing of organic and inorganic alkane gas, and the mixing of oil-formed gas and coal-formed gas, are unsuitable for the study region. Instead, we posit that tectonic activity disrupted geological structures and destroyed original shale gas reservoirs, thus leading to the escape of some gases, which then drove carbon isotope reversals in the remaining gas.
(4) The carbon isotope characteristics of normally pressured shale gas are more efficient indicators for identifying favorable shale gas sweet spots during exploration compared with geological parameters of shale samples, such as mineral composition, organic abundance, organic pore distribution, and gas content.

## Data Availability

The data that support the findings of this study are available from the corresponding authors upon reasonable request.
